# Referee acknowledgment for 2018

**DOI:** 10.1063/1.5088788

**Published:** 2019-01-30

**Authors:** Majed Chergui

The following topical experts served as referees for papers submitted to *Structural Dynamics* during 2018. The Editor-in-Chief and the Editors wish to express their appreciation to the referees for their conscientious efforts on behalf of our journal and the profession.

**A**

Abramavicius, Darius

Acremann, Yves

Adachi, Shin-Ichi

Altarelli, Massimo

**B**

Baiz, Carlos

Baldini, Edoardo

Banares, Luis

Bartlett, Rodney

Botton, Gianluigi

Bozek, John

Bredenbeck, Jens

Bressler, Christian

Brumm, Phill

**C**

Callegari, Carlo

Cao, Hongnan

Cao, Jianming

Champion, Paul

Chandrasekaran, Srinivas Niranj

Clinger, Jonathan

Cooke, David

**D**

Dawber, Matthew

Derlet, Peter

Drabbels, Marcel

**E**

Ernstorfer, Ralph

Eschenlohr, Andrea

**F**

Fuchs, Matthias

**G**

Govind, Niri

Grioni, Marco

**H**

Harbola, Upendra

Hauf, Christoph

Hoffmann, Matthias

Hommelhoff, Peter

Horn-von Hoegen, Michael

**I**

Itatani, Jiro

**J**

Johnson, Steven

**K**

Kowalewski, Markus

**L**

Laage, Damien

Laurson, Lasse

Leone, Steve

Lindenberg, Aaron

Luiten, O. J.

Lysenko, Sergiy

**M**

Mankowsky, Roman

Marangos, Jon

Meuwly, Markus

Miller, Dwayne

Miller, Mitch

**N**

Neutze, Richard

**O**

Osterwalder, Juerg

**P**

Pfau, Bastian

Puppin, Michele

**R**

Ramesh, Ramamoorthy

Rudenko, Artem

**S**

Salditt, Tim

Santra, Robin

Scholes, Greg

Scopigno, Tullio

Shepperson, Benjamin

Smirnova, Olga

Spencer, Ben F.

Springate, Emma

Stähler, Julia

Staub, Urs

Stiel, Holger

Stirnemann, Guillaume

**T**

Takeuchi, Satoshi

Temps, Friedrich

**V**

van der Veen, Renske

Vendrell, Oriol

**W**

Weissenrieder, Jonas

Wurth, Wilfried

**Y**

Yang, Shuolong

**Z**

Zhu, Yimei

